# Supplementary figures and images for: Effects of Quercetin Metabolites on Triglyceride Metabolism of 3T3-L1 Preadipocytes and Mature Adipocytes
Source: Int J Mol Sci. 2019 Jan 11;20(2):264. doi: 10.3390/ijms20020264 (PMC6359054; doi:10.3390/ijms20020264)

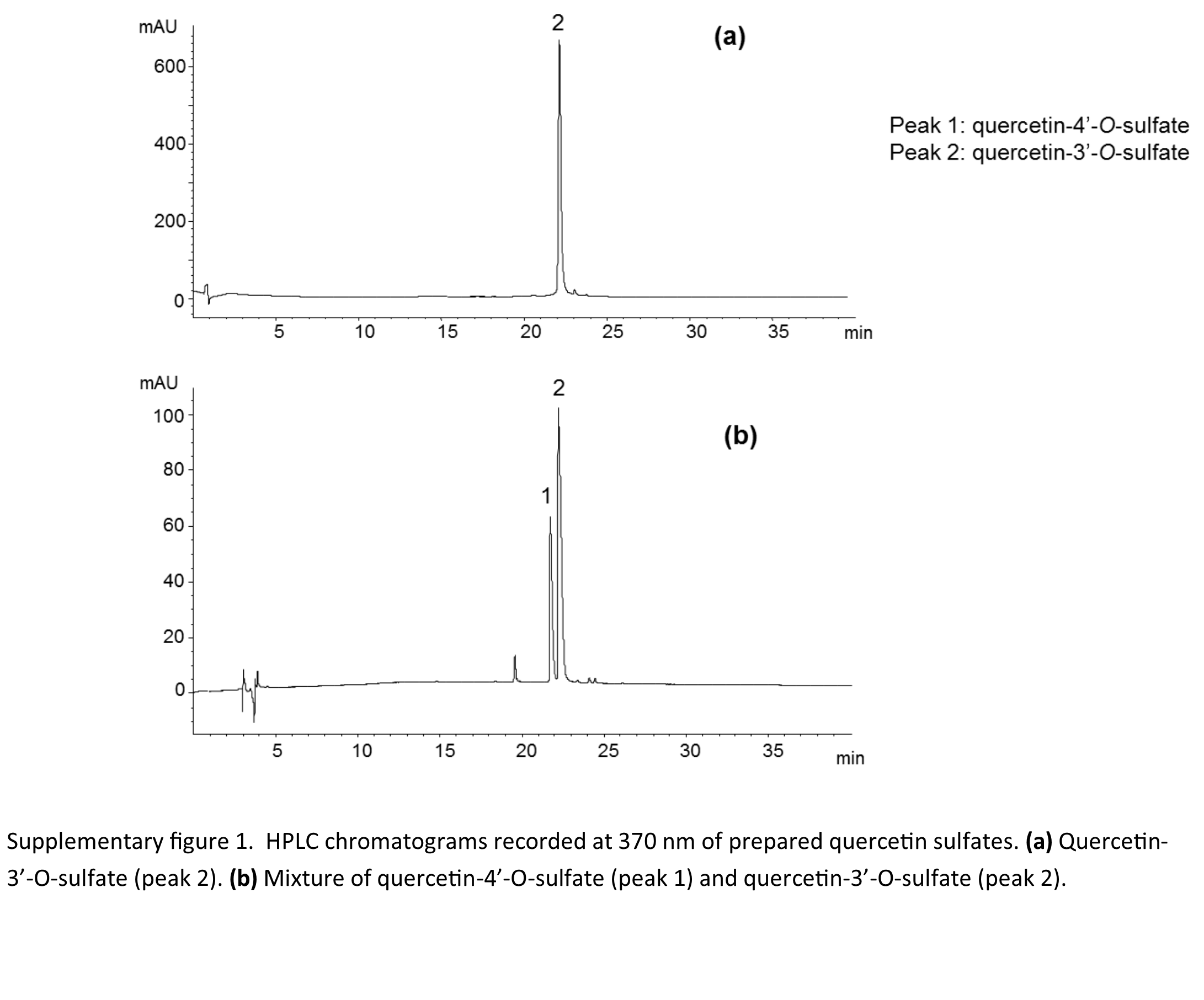

Supplement: Supplementary file 1 [file ijms-20-00264-s001.zip › Proofs supplementary/Supplementary figure 1.png]

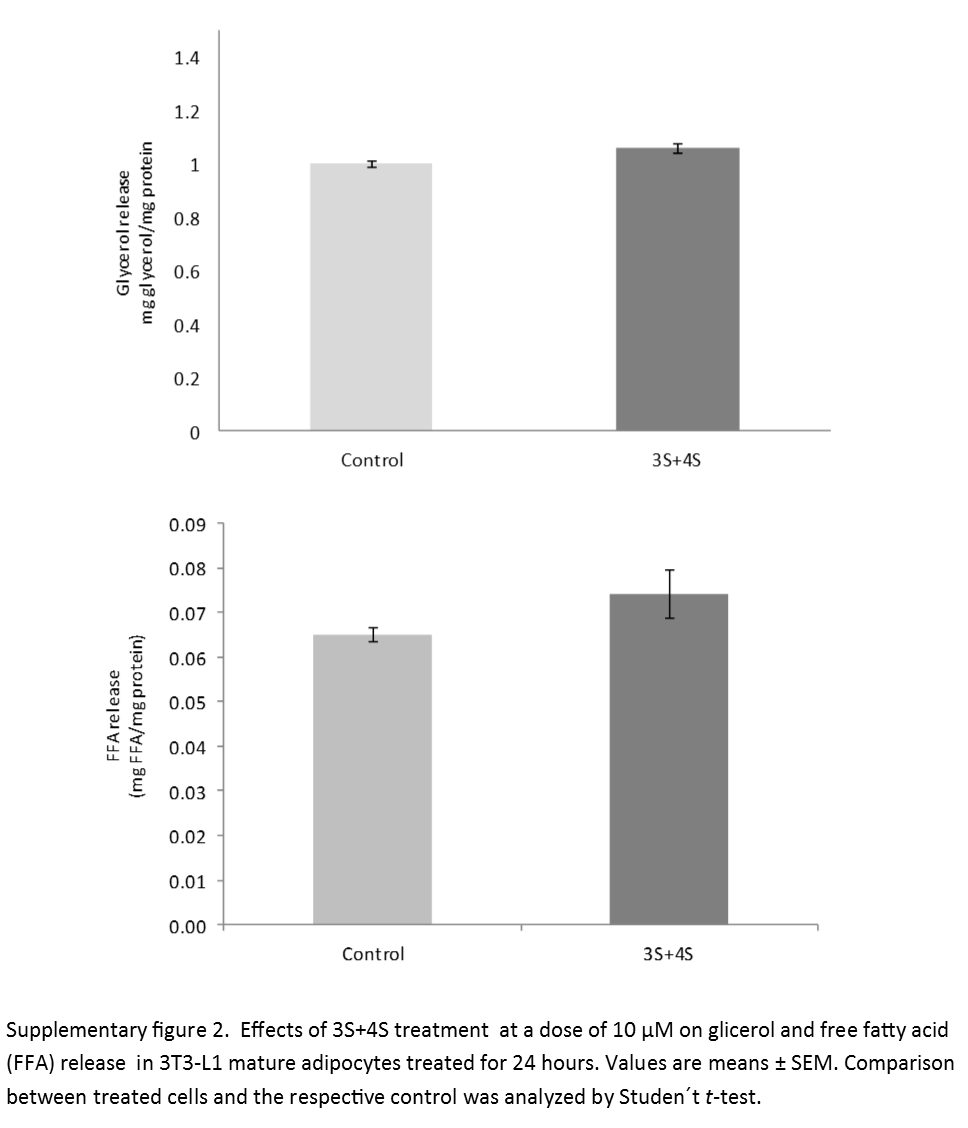

Supplement: Supplementary file 1 [file ijms-20-00264-s001.zip › Proofs supplementary/Supplementary figure 2.png]
